# Design of V-Substituted TiFe-Based Alloy for Target Pressure Range and Easy Activation

**DOI:** 10.3390/ma14174829

**Published:** 2021-08-25

**Authors:** Mohammad Faisal, June-Hyung Kim, Young Whan Cho, Jae-il Jang, Jin-Yoo Suh, Jae-Hyeok Shim, Young-Su Lee

**Affiliations:** 1 Center for Energy Materials Research, Korea Institute of Science and Technology, Seoul 02792, Korea; 120019@kist.re.kr (J.-H.K.); oze@kist.re.kr (Y.W.C.); jinyoo@kist.re.kr (J.-Y.S.); jhshim@kist.re.kr (J.-H.S.); 2 Division of Materials Science and Engineering, Hanyang University, Seoul 04763, Korea; jijang@hanyang.ac.kr

**Keywords:** hydrogen storage, TiFe alloy, equilibrium pressure, activation

## Abstract

Titanium iron (TiFe) alloy is a room-temperature hydrogen-storage material, and it absorbs hydrogen via a two-step process to form TiFeH and then TiFeH_2_. The effect of V addition in TiFe alloy was recently elucidated. The V substitution for Ti sublattice lowers *P*_2_/*P*_1_ ratio, where *P*_1_ and *P*_2_ are the equilibrium plateau pressure for TiFe/TiFeH and TiFeH/TiFeH_2_, respectively, and thus restricts the two-step hydrogenation within a narrow pressure range. The focus of the present investigation was to optimize the V content such that maximum usable storage capacity can be achieved for the target pressure range: 1 MPa for absorption and 0.1 MPa for desorption. The effect of V substitution at selective Ti or Fe sublattices was closely analyzed, and the alloy composition Ti_46_Fe_47.5_V_6.5_ displayed the best performance with ca. 1.5 wt.% of usable capacity within the target pressure range. At the same time, another issue in TiFe-based alloys, which is a difficulty in activation at room temperature, was solved by Ce addition. It was shown that 3 wt.% Ce dispersion in TiFe alloy imparted to it easy room-temperature (RT) activation properties.

## 1. Introduction

The increasing demand for an alternative to fossil fuel has stimulated hydrogen-storage research. The room-temperature hydrogen-storage alloy TiFe is the forerunner in the quest for a suitable medium owing to the abundance of constituting elements and appreciable hydrogen-storage capacity of 1.9 wt.% H [1,2] near ambient temperature and pressure. The two major bottlenecks must be overcome to commercialize TiFe alloy as a room-temperature hydrogen-storage material. First, the initial hydrogenation, or activation, should be carried out at room temperature. A high-temperature activation procedure was usually involved for activating pure TiFe [1]. Second, usable capacity under a practical operation pressure range, e.g., 1 MPa for hydrogen absorption and 0.1 MPa for desorption, must be increased. Hydrogenation of TiFe proceeds in two steps, sequentially forming TiFeH monohydride and TiFeH_2_ dihydride. The ratio of equilibrium pressure for each step, *P*_2_/*P*_1_ (*P*_1_ and *P*_2_ are the equilibrium plateau pressure for TiFe/TiFeH and TiFeH/TiFeH_2_, respectively), should be minimized to ensure large usable capacity in a narrow pressure range [2].

Available studies emphasized the characterization of the TiFe alloy, and only a few attempted to optimize the operation pressure range. Ternary phase diagram observations revealed that most of the elemental substitutions (e.g., Mn, Co, Cr, Ni, and Zr) resulted in the formation of secondary phases [3,4,5,6,7,8,9,10] dispersed uniformly in the TiFe-based primary phase [11], which was unavoidable because of the narrow single-phase region of the TiFe phase. The resultant improvement in the initial activation and other hydrogen-storage properties of TiFe alloys was attributed to the presence of these secondary hydrogen-absorbing phases [12,13]. For instance, easy activation of Zr-added TiFe alloy reported in some studies [13,14,15,16] is likely due to the formation of Ti-Fe-Zr-based ternary alloy: Faisal et al. [17] recently reported that single-phase Ti-Fe-Zr ternary alloys in the C14 structure were easily activated under 3 MPa of hydrogen pressure at room temperature. Likewise, Cr-added TiFe alloy could be activated at room temperature with the help of a Ti(Fe,Cr)_2_ phase in the C14 structure [18,19,20]. However, in those studies, the two-step plateaus became more pronounced after alloying, i.e., *P*_2_/*P*_1_ became larger. Hence, it is necessary to use alloying elements that can improve activation without modifying the thermodynamics of the TiFe phase. It was reported that over-stoichiometric Ti or the addition of mischmetal (Mm) in small quantity improved the activation properties of TiFe alloy drastically [21,22,23]. Ma et al. [24] showed that FeTi_1.3_ + x wt.% Mm (x = 1.5, 3.0, 4.5, 6.0) were hydrogenated at room temperature, with FeTi_1.3_ + 6 wt.% Mm showing 1.58 wt.% H-storage capacity. Wang et al. [22] studied hydrogen-storage properties of Ti_x_Fe + y wt.% La. La in small quantities helped improve activation of TiFe alloy without degrading hydrogen-storage capacity substantially. It has been established in previous studies [25] that the addition of a small amount of Ce showed remarkable improvement in the activation properties of TiFe_0.9_Mn_0.1_ at 353 K and 4 MPa hydrogen pressure, which was otherwise virtually impossible to activate under the above conditions [26]. Liu et al. [27] and Yan et al. [28] showed Ce segregation in Ce-containing BCC alloy. They showed that this segregation had no effect on the alloy structure and its lattice parameter. It was mentioned that Ce-containing alloys showed Ce and CeO_2_ enrichment, and Ce reacted with hydrogen, imparting RT activation properties to the alloys. Therefore, adding some percentage of Lanthanide elements can be a good strategy to solve the activation issue.

To tailor the operation pressure range of hydrogen storage via alloying, the alloy composition should stay within the single-phase TiFe in the B2 structure. It is worth mentioning that most of the element alloying with TiFe replaces Fe sublattice [15,29,30,31,32]. The conventional knowledge about Fe substitution with most-studied elements such as Ni, Co, and Cr is that they result in a significant reduction in *P*_1_, which in turn increases *P*_2_/*P*_1_ ratio. The element Mn, in contrast, leads to a lesser reduction in equilibrium pressure [26,33,34]. Liu et al. [35] showed that TiFe-based alloy demonstrated as Fe_1−x_Mn_x_Ti_1−y_V_y_ had the ability to absorb and desorb hydrogen under close-to-constant pressure, implying *P*_2_/*P*_1_ ratio approaching unity. This resulted in the enhancement of usable maximum hydrogen capacity in a narrow pressure range. Mitrokhin et al. [36] highlighted that the desorption pressure-composition isotherm curves for Ti-Fe-V-Mn alloys had the plateaus both approaching each other and being combined into one single sloping plateau. They coined the term “pressure smoothing effect” to explain the phenomenon. Massicot [37] illustrated that Ti_47_V_5_Fe_48_ displayed a sloping plateau over the complete absorption/desorption domain without a detectable two-step plateau. Jung et al. [2] pointed out the importance of V, which can substitute for both Ti and Fe sites [38] unlike the other 3*d* transition metals. This allowed more degrees of freedom in tailoring the two equilibrium pressures, *P*_1_ and *P*_2_, and an optimal hydrogen-storage property can be derived only through V without resorting to additional alloying elements.

In the current discussion, we present a systematic study of designing an optimal composition of V-substituted TiFe alloy, exhibiting *P*_2_/*P*_1_ ratio close to unity, thus maximizing the usable capacity under the target pressure range of 0.1–1 MPa. We utilized the unique property of V, i.e., its versatility to substitute for Fe and Ti, simultaneously. The role of Ce addition in improving the RT activation of V-substituted TiFe alloy was also envisaged. Thus, we propose a novel V-substituted TiFe alloy with Ce addition as a prime material sorted for RT solid-state hydrogen-storage applications.

## 2. Materials and Methods

### 2.1. Sample Synthesis

A binary Ti-Fe alloy and eight ternary Ti-Fe-V alloys with 3 wt.% Ce addition (samples 0 to 8) were synthesized via arc melting under argon (6N purity) atmosphere. The elements Ti, Fe, and V were 99.995%, 99.9%, and 99.95% pure, respectively. The required proportions of the elements were weighed such that each alloy was 25 g (±0.005 g). All the alloys were melted six times by flipping the sample button between two consecutive melting procedures to enhance the compositional homogeneity. The weight loss of the alloys after the arc melting was <0.5%. The alloys were wrapped in tantalum foil and vacuum-sealed in quartz tubes. The first six alloys (0–5) were annealed at 1000 °C for a week while the last three (6–8) were annealed for three weeks at 1000 °C. Table 1 presents nominal compositions for each alloy along with respective sample IDs. Alloy 0 is the reference alloy without V substitution. Assuming that Ti and Fe are preferentially positioned at their own sublattices, V distribution at the Ti and Fe sublattices was determined and is summarized in Table 1.

### 2.2. Structure and Chemical Composition Analysis

The crystal structure, phase fractions and lattice parameters of the alloy samples were analyzed via X-ray powder diffraction (XRD) using Cu-Kα radiation (D8 ADVANCE, Bruker AXS GmbH, Karlsruhe, Germany). Rietveld refinement analysis was performed on the diffraction patterns using TOPAS software (version 5, Bruker AXS GmbH, Karlsruhe, Germany) [39]. The phase fractions and lattice parameters were assessed via whole-profile fitting (WPF). The atomic composition of each phase was analyzed from energy-dispersive x-ray spectroscopy (EDX) on backscattered electron (BSE) images obtained from field-emission scanning electron microscope (FE-SEM, Inspect F50, FEI Company, Hillsboro, OR, USA). The phase fraction in area percentage was obtained from image analysis using Image J, FIJI, software (version 2, NIH, Bethesda, MD, USA).

### 2.3. Hydrogen Sorption Property

The alloy samples were activated in a lab-made stainless-steel reactor (3 cm^3^ capacity). The bulk alloy pieces were charged in the reactor, and the reactor was evacuated to 10^−1^ Pa at 30 °C; 3 MPa of pure hydrogen gas (6N purity) was introduced inside the reactor at room temperature, and the pressure change was monitored over time. The sample was deemed to be activated when the pressure drop inside the reactor ceased. Pressure–composition isotherm (PCI) measurement was performed on each sample to evaluate their hydrogen sorption property. Before a PCI measurement, the activated alloy sample inside the reactor was evacuated under dynamic vacuum at 200 °C for 1 h to remove any residual hydrogen from the activation process. The PCI curves were obtained at 30 °C with maximum hydrogen pressure of 4 MPa via an automatic high-pressure volumetric analyzer (HPVA II, Particulate Systems). For sample 8, PCI measurements were made at three different temperatures (30, 40, and 50 °C).

## 3. Results and Discussion

### 3.1. Alloy Design and Phase Study

**Figure 1:**
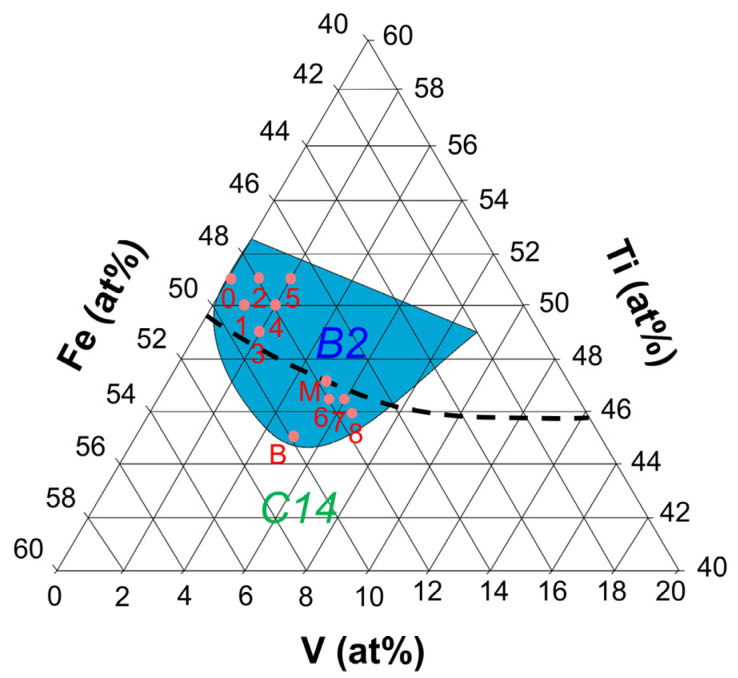
Horizontal section of a ternary phase diagram of Ti-Fe-V system at 1000 °C [38] overlaid with designed nominal compositions of samples 0–8 and the two compositions B and M from previous studies [2,38]. The shaded region corresponds to a single phase in the B2 structure. The black dashed line is a tentative liquidus surface projection and is a border between the B2 and C14 regions.

Figure 1 shows a portion of the horizontal section of a ternary phase diagram of the Ti-Fe-V system at 1000 °C (redrawn from [38]), overlaid with the nominal compositions of samples 0–8, designed for the current study. The nominal compositions from samples 0–5 were designed in a fashion that the effect of V substitution at Fe and Ti sublattices becomes evident. The composition of Ti_51_Fe_49_ (the subscript denotes the composition in at%) was chosen as the starting composition instead of Ti_50_Fe_50_ because *P*_2_ of Ti_50_Fe_50_ is higher than 1 MPa at 30 °C [1], outside the target pressure range in this study. The hydrides of Ti_51_Fe_49_ are more stable than that of Ti_50_Fe_50_. The compositions of samples 6 to 8 were designed such that the usable capacity between 0.1 and 1 MPa should be maximized: this is elaborated later.

**Figure 2:**
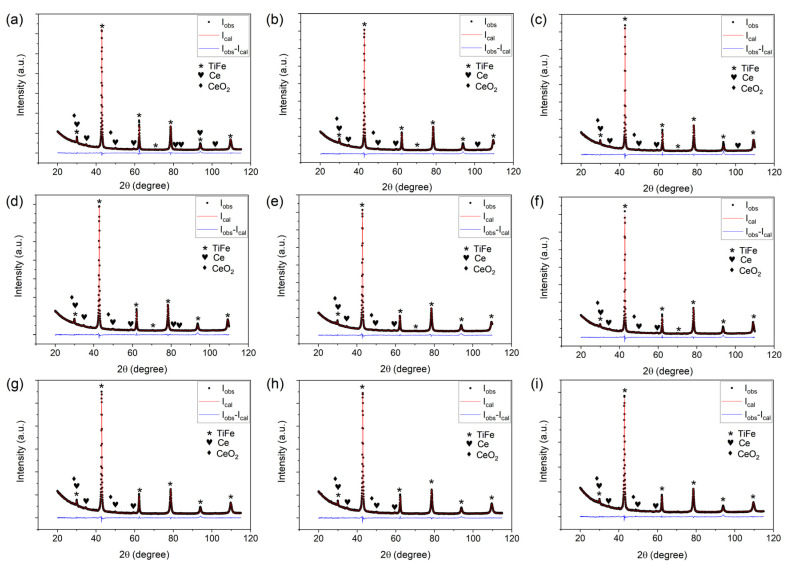
WPF plot for the XRD data from samples: (**a**) 0; (**b**) 1; (**c**) 2; (**d**) 3; (**e**) 4; (**f**) 5; (**g**) 6; (**h**) 7; (**i**) 8. The peaks of TiFe, Ce, and CeO_2_ were indexed. Observed intensity (I_obs_), calculated intensity (I_cal_), and the difference (I_obs_–I_cal_) were plotted in black circles, red line, and blue line, respectively.

The WPF plots from the XRD data of samples 0–8 are shown in Figure 2. Only three phases were detected: TiFe main phase (space group *Pm*-3*m*) and small amounts of Ce (space group *Fm*-3*m*) and CeO_2_ (space group *Fm*-3*m*) phases. Precisely speaking, the TiFe main phase is the Ti-Fe-V ternary phase in the B2 structure (in Figure 1), but we henceforth refer to it as a TiFe phase for simplicity. The Rietveld refinement results of the XRD data are summarized in Table 2. The sum of the phase fraction of Ce and CeO_2_ was about 3 wt.%, approximately the same as the initial amount of 3 wt.% Ce. Evidently, Ce is not soluble in TiFe [25] and exists as separate phases. The presence of CeO_2_ reflects that Ce acts as an oxygen scavenger, besides promoting initial hydrogenation. At 1000 °C, the standard Gibbs free energy of formation of CeO_2_ and TiO_2_ is −826 kJ mol^−1^ and −714 kJ mol^−1^, respectively (from HSC Chemistry software, version 5.1, Outokumpu Research Oy, Pori, Finland). This characteristic allows us to precisely control Ti and Fe content in the B2 structure. In a previous study, the Ti_4_Fe_2_O_1−x_ suboxide phase was found, and because of the formation of this suboxide phase, the contents of Ti and Fe were different from those of the nominal composition [2]. In the XRD data, we did not find the peaks related to this suboxide phase.

**Figure 3:**
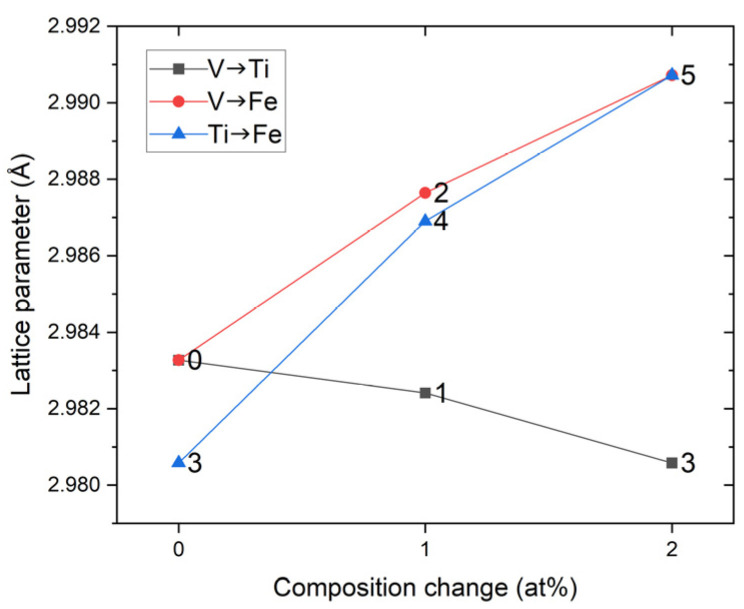
Lattice parameter change with composition for the series of the samples, 0→1→3, 0→2→5, and 3→4→5. Sample IDs are annotated. In the legend, V→Ti denotes that V replaces Ti and likewise for the other cases.

In samples 0–5, although the composition change stayed within 2 at.%, the lattice parameters in Table 2 were systematically varied according to the composition. From now on, the change in alloy composition from one sample to another is represented by ‘→’. For instance, if the composition of sample 0 changes to 1, it would be represented as 0→1. Table 2 shows that samples 0→1→3 have Fe composition fixed to 49 at.%, with increasing V in 0→1→2 at.% and decreasing Ti in 51→50→49 at.%. The atomic radii for the elements are in the following order: Ti > V > Fe. Therefore, when V substitutes for Ti (V→Ti), the lattice parameter must decrease. This trend is illustrated in Figure 3. Similarly, samples 0→2→5 have Ti composition fixed at 51 at% with V substituting for Fe (V→Fe). The lattice parameter monotonically increased in this series. For samples 3→4→5, Ti substituted for Fe (Ti→Fe), and again the lattice parameter increased showing the largest slope among the three series in Figure 3.

**Figure 4:**
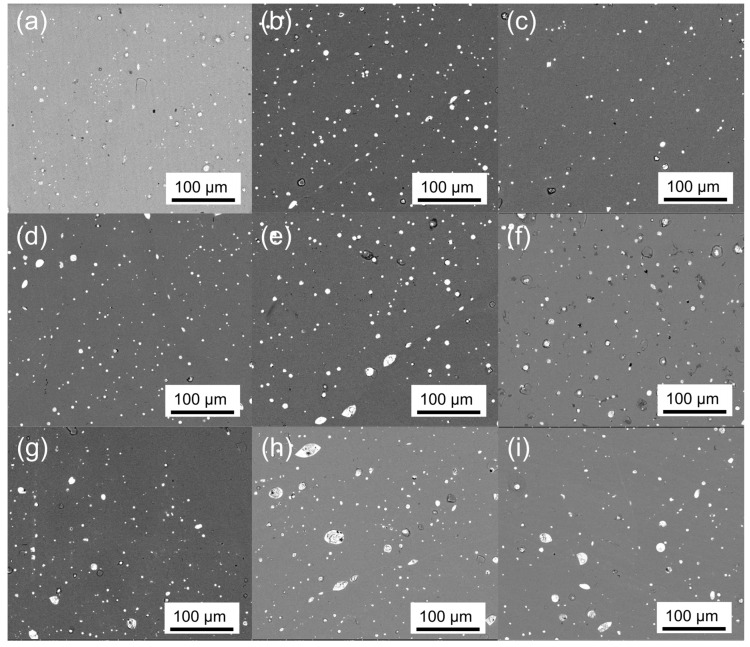
SEM BSE images of samples: (**a**) 0; (**b**) 1; (**c**) 2; (**d**) 3; (**e**) 4; (**f**) 5; (**g**) 6; (**h**) 7; (**i**) 8.

**Figure 5:**
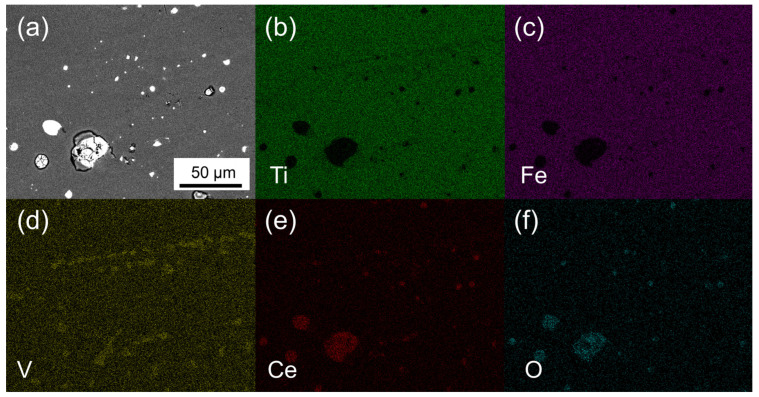
SEM BSE image and EDX elemental map of sample 8: (**a**) BSE image; (**b**) Ti; (**c**) Fe; (**d**) V; (**e**) Ce; (**f**) O.

BSE images of the samples in Figure 4 further validated the phase analysis from XRD. In Figure 4, only two contrasts were evident regardless of the sample ID. The EDX elemental map of sample 8 shown in Figure 5 revealed that the gray or dark contrast was the TiFe phase. The white or bright contrast showed enrichment of Ce and O, and they corresponded to phases Ce and/or CeO_2_. The area fractions evaluated for gray and white contrast are summarized in Table 3 and in good agreement with the phase fractions from XRD data in Table 2. Thus, the above observation suggests that phases and their relative fractions obtained after melting and heat treatment were in confirmation with the respective intentionally designed alloys.

### 3.2. Activation and PCI Measurements

**Figure 6:**
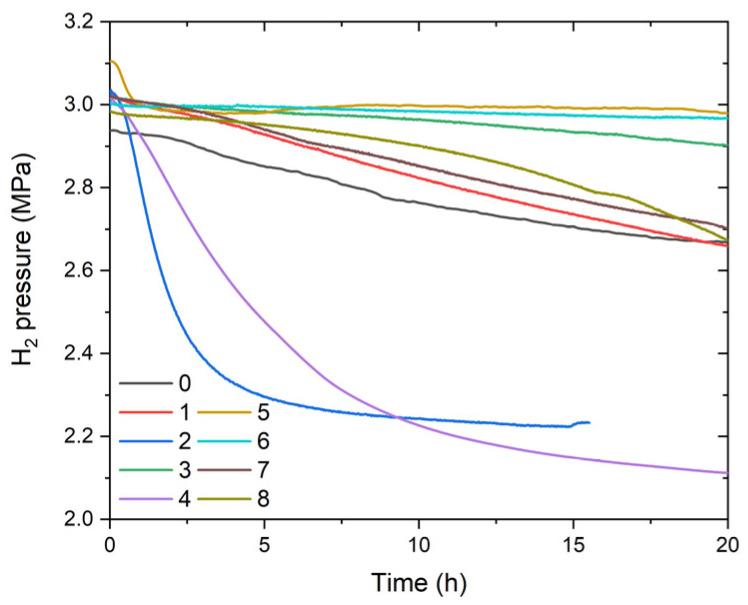
First-cycle activation profile for samples 0–8. The hydrogen pressure inside the closed reactor was recorded for 20 h.

Activation of alloys 0–8 was performed at 30 °C under 3 MPa H_2_ pressure. As shown in Figure 6, all the alloys absorbed hydrogen under the above condition although the kinetics varied among the samples. The effect of Ce addition to TiFe-based alloys has been highlighted earlier [25], and also in our cases, the addition of 3 wt.% Ce promoted activation. Complete activation of each alloy took three to five cycles of hydrogen absorption at 3 MPa followed by evacuation under rough vacuum (10^−1^ Pa) at 30 °C.

**Figure 7:**
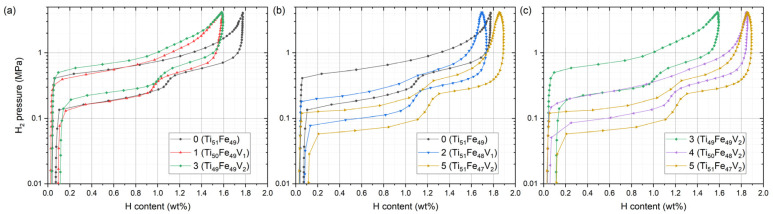
PCI curves for the three series of the samples measured at 30 °C: (**a**) 0, 1, and 3; (**b**) 0, 2, and 5; (**c**) 3, 4, and 5.

In Figure 7, the PCI curves for the three series of the composition change are presented: 0→1→3, 0→2→5, and 3→4→5, as the lattice parameter analysis in Figure 3. It can be seen that the desorption plateau pressures *P*_1_ and *P*_2_ for the starting composition 0 were positioned within 0.1–1 MPa, but pressure hysteresis in the absorption process limited the absorbed amount under 1 MPa, and further optimization of the composition was necessary. When comparing the plateau pressures, we focused on the desorption plateau pressures because the pressure hysteresis in Figure 7 was apparently affected by the composition and the absorption plateau pressures were less systematically changed by the composition change.

**Figure 8:**
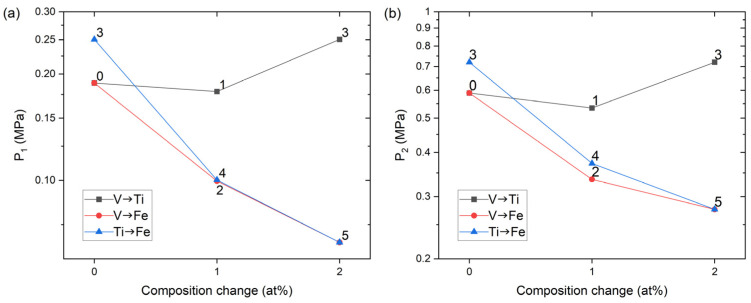
Variation of the desorption plateau pressures (**a**) *P*_1_ and (**b**) *P*_2_ for the three series of the samples in Figure 7. Sample IDs are annotated. In the legend, V→Ti denotes that V replaces Ti and likewise for the other cases.

We start the discussion with sample 0→1→3 in Figure 7a. As the composition of the alloy sample changed 0→1, there was no profound change in the plateau pressure. In contrast, with increasing V from 1 to 2 at.%, in 1→3, *P*_1_ and *P*_2_ certainly rose to higher pressures. Referring to the sublattice occupation in Table 1, Ti at Fe sublattice was replaced by V in 0→1 (Fe_49_Ti_1_→ Fe_49_V_1_), whereas Ti at Ti sublattice was replaced by V in 1→3 (Ti_50_→Ti_49_V_1_). Therefore, equilibrium pressure tailoring outcomes would differ depending on the V site occupancy [2]: Ti or V substitution at Fe sublattice changed the plateau pressure to a similar degree, but V substitution at Ti sublattice raised the plateau pressures. The PCI curves of the second series 0→2→5, plotted in Figure 7b, displayed a monotonic decrease in the plateau pressures. Different from the aforementioned 0→1→3 series, here only Fe at Fe sublattice was replaced by V as V concentration increased from 0 to 2 at.%. In the last series, 3→4→5 in Figure 7c, *P*_1_ and *P*_2_ again decreased monotonically. However, the drop in 3→4 was more pronounced than that in 4→5. This is also related to the underlying substitution mechanism in Table 1. At the Fe sublattice, the occupation change in 3→4 (Fe_49_V_1_→Fe_48_V_2_) and 4→5 (Fe_48_V_2_→Fe_47_Ti_1_V_2_) was the increase of either V or Ti by 1 at.%, and we found that the effect on the pressure change was similar. However, there was an additional change in the Ti sublattice occupation in 3→4 (Ti_49_V_1_→Ti_50_), and this resulted in a more significant drop in *P*_1_ and *P*_2_ in the case of 3→4. Therefore, the plateau pressure change is comprehensible only when the sublattice occupation is considered [40]: the nominal composition change does not provide full understanding. To clearly visualize the pressure variation, *P*_1_ and *P*_2_ were taken at the midpoint of the desorption plateaus and are plotted in Figure 8.

Based on the PCI results, we established a strategy to maximize the usable capacity between 0.1 and 1 MPa: *P*_2_ is lowered through V substitution at Fe sublattice, and then *P*_1_ is raised by V substitution at Ti sublattice. In Figure 8, *P*_2_ of the samples 2, 4, and 5 is low enough to ensure a high amount of hydrogen absorption under 1 MPa even with pressure hysteresis. Therefore 2–3 at.% V at Fe sublattice can bring down the *P*_2_ value to the desired pressure range. However, then their *P*_1_ values become too low to fully desorb hydrogen under 0.1 MPa. Considering the increase in *P*_1_ in 1→3 when 1 at.% of V replaced Ti, 3–4 at.% V at Ti sublattice would be needed to make *P*_1_ similar to *P*_2_. Under this logic, we designed three new compositions: Ti_46.5_Fe_48_V_5.5_ (sample 6), Ti_46.5_Fe_47.5_V_6_ (sample 7), and Ti_46_Fe_47.5_V_6.5_ (sample 8). A precaution was taken when preparing these samples. In Figure 1, the black dashed line is the liquidus surface projection, overlaid on a portion of the isothermal section of the Ti-Fe-V phase diagram at 1000 °C [38]. The alloy composition marked as B (Ti_45_Fe_50_V_5_) is located in the region where C14 is solidified first from liquid. This composition contained ~10 wt.% of the C14 phase even after 1 week of annealing in a previous study [2], and it would require a longer annealing time to reach thermodynamic equilibrium, i.e., a single B2 phase. It was shown in another study that the alloy composition marked as M (Ti_47_V_5_Fe_48_), which is on the liquidus surface projection line that divides the region of B2 and C14, resulted in an almost-B2 phase after three weeks of annealing [37]. Therefore, samples 6–8 need a longer annealing time than that applied for samples 0–5 (one week), and they were annealed for three weeks at 1000 °C. The XRD data in Figure 2g–i for samples 6–8 confirmed that they only contained the TiFe phase in the B2 structure except for Ce and CeO_2_; we did not find the peaks of the C14 phase.

**Figure 9:**
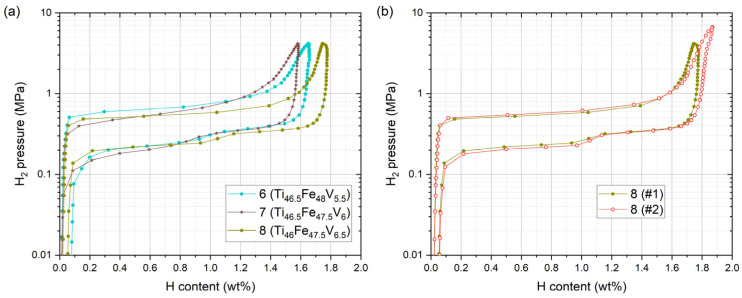
(**a**) PCI curves for samples 6, 7, and 8 at 30 °C. (**b**) Comparison of the two independently measured PCI curves for sample 8.

Figure 9a shows the PCI curves for samples 6, 7, and 8 measured at 30 °C. As intended, *P*_1_ and *P*_2_ were located closely for all of them, and the two-step desorption plateaus became almost a single plateau. Although it is a subtle difference, *P*_1_ of sample 7 was the lowest because it contained more V at Fe sublattice than sample 6 and less V at Ti sublattice than sample 8; this again proves that the equilibrium pressure can be precisely engineered if the effect of substitution at each sublattice is well understood. All of them turned out to be suitable to be used between the target pressure range of 0.1–1 MPa. Among them, sample 8 displayed the best performance. Although further analyses are necessary, it is also possible that the smaller maximum hydrogen content in samples 6 and 7 could have originated from the failure of full activation of the samples rather than from the difference in composition. Samples 1 and 3 in Figure 7a also displayed smaller maximum capacities, and there is no apparent reason why such a small change in composition can significantly affect the maximum capacity. Their PCI curves look squeezed along the abscissa, and it indicates that some part of the samples remained unactivated. To determine the reproducibility of the PCI curve of sample 8, another measurement was performed and presented as #2 in Figure 9b. The data were not from the consecutive cycle of #1 but an independent measurement with a new sampling and the activation process. 

Continuing the discussion on the usable capacity, sample 8, showing the best performance, absorbed ~1.6 wt.% H under 1 MPa and desorbed most of the absorbed hydrogen at 0.1 MPa, resulting in 1.5 wt.% of usable hydrogen-storage capacity in this range. This surpasses the maximum capacity of 1.4 wt.% of LaNi_5_ (LaNi_5_ to LaNi_5_H_6_) and highlights the potential of TiFe-based alloy as a room-temperature hydrogen-storage material. Further improvement in the usable capacity by increasing V content is conceivable. However, as shown in Figure 1, composition 8 is close to the phase boundary of the B2 single phase, and the V content is limited by thermodynamics. Therefore we do not expect any significant improvement in the usable capacity by further increasing V content. 

**Figure 10:**
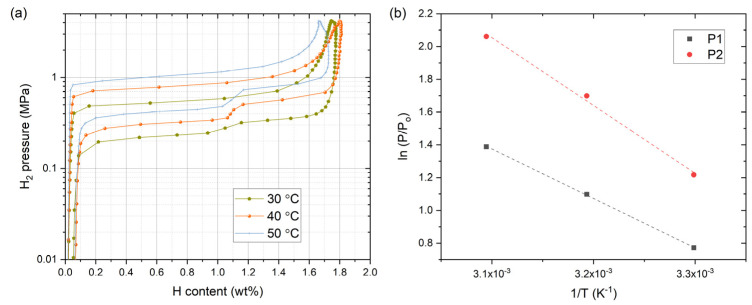
(**a**) PCI curves for sample 8 at 30, 40, and 50 °C; (**b**) van’t Hoff plots of the desorption plateau pressures for sample 8. The dashed lines in (**b**) are the linear regression results and *P*_0_ = 1.01325 × 10^5^ Pa.

The enthalpy and entropy change of hydrogen desorption of sample 8 was characterized by measuring PCI curves at 40 and 50 °C, and the results are shown in Figure 10a. The *P*_1_ and *P*_2_ from the desorption plateaus were taken at H/M = 0.25 (0.486 wt.% H) and H/M = 0.7 (1.361 wt.% H), respectively, where H/M is the hydrogen to metal ratio, and van’t Hoff plots are shown in Figure 10b. The enthalpy change (∆*H*) and entropy change (∆*S*) per one mole of hydrogen were obtained from the slope and the intercept of the linear regression result. The obtained values are: ∆*H*_1_ = 25.1 ± 0.4 kJ mol^−1^ and ∆*H*_2_ = 34.4 ± 2.2 kJ mol^−1^, and ∆*S*_1_ = 89.3 ± 1.2 J K^−1^ mol^−1^ and ∆*S*_2_ = 123.8 ± 7.0 J K^−1^ mol^−1^. Compared to the enthalpy change of pure TiFe, ∆*H*_1_ = 28.1 kJ mol^−1^ and ∆*H*_2_ = 33.3 kJ mol^−1^ (at H/M = 0.7) by Reilly and Wiswall [1] and ∆*H*_1_ = 25.6 ± 1.0 and ∆*H*_2_ = 31.6 ± 1.2 kJ mol^−1^ (at H/M = 0.7) by Wenzl and Lebsanft [41], ∆*H*_1_ was down-shifted and ∆*H*_2_ was up-shifted. Especially, the difference, ∆*H*_2_−∆*H*_1_, was appreciably larger in sample 8. Hence the decrease in *P*_2_ (i.e., stabilization of dihydride and larger ∆*H*_2_) and the increase in *P*_1_ (i.e., destabilization of monohydride and smaller ∆*H*_1_) by V substitution were correctly captured in the enthalpy change.

## 4. Conclusions

The objectives of the present investigation were twofold: (1) design of a TiFe alloy with a maximum usable hydrogen-storage capacity in a target pressure range (0.1–1 MPa) and (2) easy RT activation of the TiFe alloy via Ce addition. Enhancement in the usable storage capacity was achieved via V substitution. The unique characteristic of V, i.e., appreciable substitution at both Ti and Fe sublattices, was exploited to tailor the plateau pressures. The cue to enhance hydrogen-storage capacity is the reduction in the *P*_2_/*P*_1_ ratio: *P*_2_ was lowered by V substitution at Fe sublattice whereas *P*_1_ was raised by V substitution at Ti sublattice. The addition of 3 wt.% Ce promoted activation at room temperature and suppressed the formation of Ti_4_Fe_2_O_1−x_ suboxide, which enabled better control of the composition of the TiFe phase. Among the series of the Ti-Fe-V alloys, composition Ti_46_Fe_47.5_V_6.5_ exhibited the maximum usable capacity of 1.5 wt.% under 1 MPa of absorption and 0.1 MPa of desorption pressure at 30 °C. The enthalpy change of hydrogen desorption was ∆*H*_1_ = 25.1 ± 0.4 kJ mol^−1^ and ∆*H*_2_ = 34.4 ± 2.2 kJ mol^−1^, providing an increased value of ∆*H*_2_−∆*H*_1_ compared to pure TiFe.

## Figures and Tables

**Table 1:** Nominal composition of the samples from 0 to 8. The subscripts indicate at% of the corresponding elements. Third and fourth columns show atom distribution at the Ti and Fe sublattices.

Sample ID	Nominal Composition	Ti Sublattice	Fe Sublattice
0	Ti_51_Fe_49_	Ti_50_	Fe_49_Ti_1_
1	Ti_50_Fe_49_V_1_	Ti_50_	Fe_49_V_1_
2	Ti_51_Fe_48_V_1_	Ti_50_	Fe_48_Ti_1_V_1_
3	Ti_49_Fe_49_V_2_	Ti_49_V_1_	Fe_49_V_1_
4	Ti_50_Fe_48_V_2_	Ti_50_	Fe_48_V_2_
5	Ti_51_Fe_47_V_2_	Ti_50_	Fe_47_Ti_1_V_2_
6	Ti_46.5_Fe_48_V_5.5_	Ti_46.5_V_3.5_	Fe_48_V_2_
7	Ti_46.5_Fe_47.5_V_6_	Ti_46.5_V_3.5_	Fe_47.5_V_2.5_
8	Ti_46_Fe_47.5_V_6.5_	Ti_46_V_4_	Fe_47.5_V_2.5_

**Table 2:** XRD analysis of the samples. The lattice parameter of the TiFe main phase and the phase fractions determined by the Rietveld refinement of the XRD data in Figure 2. The weighted profile *R*-factor (*R*_wp_) and the goodness of fit (χ^2^) are listed to show the quality of the fit.

Sample ID	Nominal Composition	Lattice Parameter (Å)	Phase Fraction (wt.%)			*R*_wp_ (%)	χ^2^
			TiFe	Ce	CeO_2_		
0	Ti_51_Fe_49_	2.98327 (5)	96.9 (2)	1.3 (1)	1.8 (2)	3.52	2.83
1	Ti_50_Fe_49_V_1_	2.98241 (7)	97.2 (2)	1.4 (1)	1.4 (2)	3.41	2.15
2	Ti_51_Fe_48_V_1_	2.98764 (6)	97.2 (3)	1.1 (1)	1.7 (3)	4.07	2.57
3	Ti_49_Fe_49_V_2_	2.98058 (7)	97.5 (2)	0.9 (1)	1.6 (1)	3.09	2.00
4	Ti_50_Fe_48_V_2_	2.98690 (6)	96.8 (3)	1.3 (1)	1.9 (3)	3.28	2.10
5	Ti_51_Fe_47_V_2_	2.99072 (6)	97.5 (2)	1.0 (1)	1.5 (1)	3.54	2.21
6	Ti_46.5_Fe_48_V_5.5_	2.98008 (6)	96.7 (2)	1.2 (1)	2.1 (2)	3.29	1.93
7	Ti_46.5_Fe_47.5_V_6_	2.98179 (6)	96.9 (2)	1.0 (1)	2.1 (2)	3.04	1.79
8	Ti_46_Fe_47.5_V_6.5_	2.98122 (7)	96.5 (2)	1.1 (1)	2.4 (2)	3.26	1.89

**Table 3:** Phase analysis of the samples from the SEM micrographs. The phase fractions from the BSE images.

Sample ID	Nominal Composition	Phase Fraction (Area%)	
		Gray	White
0	Ti_51_Fe_49_	96.9	3.1
1	Ti_50_Fe_49_V_1_	96.9	3.1
2	Ti_51_Fe_48_V_1_	97.4	2.6
3	Ti_49_Fe_49_V_2_	97.0	3.0
4	Ti_50_Fe_48_V_2_	96.5	3.5
5	Ti_51_Fe_47_V_2_	97.2	2.8
6	Ti_46.5_Fe_48_V_5.5_	97.0	3.0
7	Ti_46.5_Fe_47.5_V_6_	96.8	3.2
8	Ti_46_Fe_47.5_V_6.5_	97.0	3.0

## Data Availability

Not applicable.

## References

[1] Reilly J.J., Wiswall R.H. (1974). Formation and properties of iron titanium hydride. Inorg. Chem..

[2] Jung J.Y., Lee Y.S., Suh J.Y., Huh J.Y., Cho Y.W. (2021). Tailoring the equilibrium hydrogen pressure of TiFe via vanadium substitution. J. Alloys Compd..

[3] Lee S.M., Perng T.P. (1999). Correlation of substitutional solid solution with hydrogenation properties of TiFe_1-x_M_x_ (M = Ni, Co, Al) alloys. J. Alloys Compd..

[4] Bououdina M., Fruchart D., Jacquet S., Pontonnier L., Soubeyroux J.L. (1999). Effect of nickel alloying by using ball milling on the hydrogen absorption properties of TiFe. Int. J. Hydrogen Energy.

[5] Lee S.M., Perng T.P. (1994). Effect of the second phase on the initiation of hydrogenation of TiFe_1−x_M_x_ (M = Cr, Mn) alloys. Int. J. Hydrogen Energy.

[6] Oguro K., Osumi Y., Suzuki H., Kato A., Imamura Y., Tanaka H. (1983). Hydrogen storage properties of TiFe_1−x_Ni_y_M_z_ alloys. J. Less-Common. Met..

[7] Qu H., Du J., Pu C., Niu Y., Huang T., Li Z., Lou Y., Wu Z. (2015). Effects of Co introduction on hydrogen storage properties of Ti–Fe–Mn alloys. Int. J. Hydrogen Energy.

[8] Patel A.K., Duguay A., Tougas B., Neumann B., Schade C., Sharma P., Huot J. (2021). Study of the Microstructural and First Hydrogenation Properties of TiFe Alloy with Zr, Mn and V as Additives. Processes.

[9] Dematteis E.M., Dreistadt D.M., Capurso G., Jepsen J., Cuevas F., Latroche M. (2021). Fundamental hydrogen storage properties of TiFe-alloy with partial substitution of Fe by Ti and Mn. J. Alloys Compd..

[10] Dematteis E.M., Berti N., Cuevas F., Latroche M., Baricco M. (2021). Substitutional effects in TiFe for hydrogen storage: A comprehensive review. Mater. Adv..

[11] Zeng L., Xu G., Liu L., Bai W., Zhang L. (2018). Experimental investigation of phase equilibria in the Ti-Fe-Zr system. Calphad.

[12] Gosselin C., Santos D., Huot J. (2017). First hydrogenation enhancement in TiFe alloys for hydrogen storage. J. Phys. D Appl. Phys.

[13] Lv P., Huot J. (2016). Hydrogen storage properties of Ti_0.95_FeZr_0.05_, TiFe_0.95_Zr_0.05_ and TiFeZr_0.05_ alloys. Int. J. Hydrogen Energy.

[14] Gosselin C., Huot J. (2015). Hydrogenation Properties of TiFe Doped with Zirconium. Materials.

[15] Jain P., Gosselin C., Huot J. (2015). Effect of Zr, Ni and Zr_7_Ni_10_ alloy on hydrogen storage characteristics of TiFe alloy. Int. J. Hydrogen Energy.

[16] Lv P., Huot J. (2017). Hydrogenation improvement of TiFe by adding ZrMn_2_. Energy.

[17] Faisal M., Suh J.Y., Lee Y.S. (2021). Understanding first cycle hydrogenation properties of Ti–Fe–Zr ternary alloys. Int. J. Hydrogen Energy.

[18] Kim H., Faisal M., Lee S.I., Jung J.Y., Kim H.J., Hong J., Lee Y.S., Shim J.H., Cho Y.W., Kim D.H. (2021). Activation of Ti–Fe–Cr alloys containing identical AB_2_ fractions. Alloy. Compd..

[19] Jung J.Y., Lee S.I., Faisal M., Kim H., Lee Y.S., Suh J.Y., Shim J.H., Huh J.Y., Cho Y.W. (2021). Effect of Cr addition on room temperature hydrogenation of TiFe alloys. Int. J. Hydrogen Energy.

[20] Park K.B., Ko W.S., Fadonougbo J.O., Na T.W., Im H.T., Park J.Y., Kang J.W., Kang H.S., Park C.S., Park H.K. (2021). Effect of Fe substitution by Mn and Cr on first hydrogenation kinetics of air-exposed TiFe-based hydrogen storage alloy. Mater. Charact..

[21] Bronca V., Bergman P., Ghaemmaghami V., Khatamian D., Manchester F.D. (1985). Hydrogen absorption characteristics of an FeTi + misch metal alloy. J Less-Common Met..

[22] Wang X., Chen R., Chen C., Wang Q. (2006). Hydrogen storage properties of Ti_x_Fe + y wt.% La and its use in metal hydride hydrogen compressor. J. Alloys Compd..

[23] Singh B.K., Singh A.K., Srivastava O.N. (1996). Improved hydrogen sorption characteristics in FeTi_1 + x_Mm material. Int. J. Hydrogen Energy.

[24] Ma J., Pan H., Wang X., Chen C., Wang Q. (2000). Hydrogen storage properties of FeTi_1.3_+x wt%Mm (x=0.0, 1.5, 3.0, 4.5, 6.0) hydrogen storage alloys. Int. J. Hydrogen Energy.

[25] Leng H., Yu Z., Yin J., Li Q., Wu Z., Chou K.-C. (2017). Effects of Ce on the hydrogen storage properties of TiFe_0.9_Mn_0.1_ alloy. Int. J. Hydrogen Energy.

[26] Nagai H., Kitagaki K., Shoji K. (1987). Microstructure and hydriding characteristics of FeTi alloys containing manganese. J. Less-Common Met..

[27] Liu X.P., Cuevas F., Jiang L.J., Latroche M., Li Z.N., Wang S.M. (2009). Improvement of the hydrogen storage properties of Ti–Cr–V–Fe BCC alloy by Ce addition. J. Alloys Compd..

[28] Yan Y., Chen Y., Liang H., Zhou X., Wu C., Tao M. (2007). Effect of Ce on the structure and hydrogen storage properties of V_55_Ti_22.5_Cr_16.1_Fe_6.4_. J. Alloys Compd..

[29] Bruzzone G., Costa G., Ferretti M., Olcese G.L. (1980). Hydrogen storage in a beryllium substituted TiFe compound. Int. J. Hydrogen Energy.

[30] Yang T., Wang P., Xia C., Liu N., Liang C., Yin F., Li Q. (2020). Effect of chromium, manganese and yttrium on microstructure and hydrogen storage properties of TiFe-based alloy. Int. J. Hydrogen Energy.

[31] Zadorozhnyy V.Y., Klyamkin S.N., Zadorozhnyy M.Y., Bermesheva O.V., Kaloshkin S.D. (2014). Mechanical alloying of nanocrystalline intermetallic compound TiFe doped by aluminum and chromium. J. Alloys Compd..

[32] Berdonosova E.A., Zadorozhnyy V.Y., Zadorozhnyy M.Y., Geodakian K.V., Zheleznyi M.V., Tsarkov A.A., Kaloshkin S.D., Klyamkin S.N. (2019). Hydrogen storage properties of TiFe-based ternary mechanical alloys with cobalt and niobium. A thermochemical approach. Int. J. Hydrogen Energy.

[33] Johnson J.R., Reilly J.J. The Use of Manganese Substituted Ferrotitanium Alloys for Energy Storage. Proceedings of the International Conference on Alternative Energy Sources.

[34] Mintz M.H., Vaknin S., Biderman S., Hadari Z. (1981). Hydrides of ternary TiFe_x_M_1−x_ (M=Cr, Mn, Co, Ni) intermetallics. J. Appl. Phys.

[35] Liu J., Lundin C.E. (1982). Alloys for Hydrogen Storage.

[36] Mitrokhin S.V., Verbetsky V.N., Kajumov R.R., Cunmao H., Yufen Z. (1993). Hydrogen sorption peculiarities in FeTi-type Ti-Fe-V-Mn alloys. J. Alloys Compd..

[37] Massicot B. (2009). Étude du Système Fe–Ti–V et de ses Applications au Stockage de Lߣhydrogène. Ph.D. Thesis.

[38] Massicot B., Joubert J.M., Latroche M. (2010). Phase equilibria in the Fe–Ti–V system. Int. J. Mat. Res..

[39] (2014). TOPAS Version 5.

[40] Ko W.S., Park K.B., Park H.K. (2021). Density functional theory study on the role of ternary alloying elements in TiFe-based hydrogen storage alloys. J. Mater. Sci. Technol..

[41] Wenzl H., Lebsanft E. (1980). Phase diagram and thermodynamic parameters of the quasibinary interstitial alloy Fe_0.5_Ti_0.5_H_x_ in equilibrium with hydrogen gas. J. Phys. F Met. Phys.

